# Application of ultrasound-guided percutaneous catheter drainage in the treatment of tuberculous psoas abscesses

**DOI:** 10.3389/fmed.2026.1776413

**Published:** 2026-04-29

**Authors:** Yongjie Yu, Yiting Guo, Dan Zhao, Zhen Lai, Yinan Dong

**Affiliations:** 1 Department of Osteology, Hangzhou Red Cross Hospital, Tuberculosis Diagnostic and Treatment Center of Zhejiang Province, Hangzhou, Zhejiang, China; 2 Department of Ultrasonography, The Second Clinical Medical College, Zhejiang Chinese Medical University, Hangzhou, China; 3 Department of Ultrasonography, Hangzhou Red Cross Hospital, Tuberculosis Diagnostic and Treatment Center of Zhejiang Province, Hangzhou, Zhejiang, China

**Keywords:** percutaneous catheter drainage, psoas abscesses, treatment, tuberculous, ultrasound-guided

## Abstract

**Objective:**

To evaluate the clinical utility of ultrasound-guided percutaneous catheter drainage (UPCD) in treating tuberculous psoas abscesses (TPA).

**Methods:**

A retrospective analysis was conducted on 114 cases of TPA with anti-tuberculosis chemotherapy at our institution. The patients were categorized into three groups based on their treatment modalities: preoperative UPCD combined with surgery (43 cases), surgery-only (39 cases), and UPCD-only (32 cases). Surgical debridement time and blood loss were compared between the preoperative UPCD combined with surgery group and the surgery-only group. Linear regression analysis was employed to evaluate the correlation between abscess volume and both debridement time and blood loss. Furthermore, recurrence and sinus tract formation were assessed across the groups during a 24-month follow-up period.

**Results:**

The preoperative UPCD combined with surgery group had significantly shorter debridement times and less blood loss compared to the surgery-only group (26.30 ± 4.14 min *vs.* 34.18 ± 6.30 min, *p* < 0.01; 156.05 ± 39.47 mL *vs.* 237.05 ± 78.18 mL, *p* < 0.01). Linear regression analysis revealed a positive correlation between abscess volume and debridement time in both groups, although the Coefficient B in the preoperative UPCD combined with surgery group was significantly lower than that in the surgery-only group. In the surgery-only group, blood loss was positively correlated with abscess volume, whereas no such correlation was found in the preoperative UPCD combined with surgery group.

**Conclusion:**

UPCD demonstrated substantial clinical value in the management of TPA. This approach not only reduced surgical time and blood loss but also provided a minimally invasive therapeutic option, especially when combined with anti-tuberculosis chemotherapy.

## 1 Introduction

Tuberculosis continues to be a significant global health challenge, affecting millions of individuals and resulting in a considerable number of deaths annually (1). Tuberculous psoas abscess (TPA) is a relatively common form of extra-pulmonary tuberculosis in regions where the disease is endemic (2). It is caused by infection with *Mycobacterium tuberculosis* and is associated with spinal tuberculosis (Pott’s disease) in approximately 75 ~ 83% of cases (3). Treatment is challenging, with a high propensity for recurrence or sinus tract formation (4).

It is widely recognized that early, continuous, and combination anti-tuberculosis chemotherapy administered at appropriate doses is the most effective strategy for treating tuberculosis (5). However, for localized lesions such as thick-walled tuberculosis abscesses, where drug concentrations within the lesion fail to reach therapeutic levels, conventional anti-tuberculosis chemotherapy only often proves inadequate (6). Surgical intervention, therefore, is considered an effective local management option, and when combined with anti-tuberculosis chemotherapy, it can significantly expedite the healing process (7, 8). Traditional surgical techniques, such as incision and drainage, though effective, are associated with substantial surgical trauma, a higher incidence of complications, and prolonged patient recovery times (9, 10).

In recent years, with the advancement of ultrasound technology, ultrasound-guided percutaneous catheter drainage (UPCD) has emerged as a novel approach for treating deep abscesses (11). However, reports on its application in the management of TPA remain relatively scarce. This study aims to evaluate the efficacy of UPCD in TPA retrospectively and to assess its clinical utility.

## 2 Methods

### 2.1 Patients

This study was approved by the Medical Ethics Committee of Hangzhou Red Cross Hospital (No. 2021–298) and comprised a retrospective analysis of patients who received initial treatment for TPA at our institution between January 2021 and December 2022, conducted in full accordance with the STROBE guidelines. Simultaneously, the orthopedic surgeons performing debridement, the radiologists administering UPCD, the tuberculosis specialists involved in medical treatment, and all other participating clinicians were unaware of the research methodology and objectives during patient treatment. Ultimately, the data were collected by physicians not directly involved in patient care, based on the content of medical records and follow-up results, thereby minimizing the potential influence of subjective factors on the outcomes. Informed consent was obtained from all patients, and all cases were rigorously confirmed through pathological or laboratory diagnostics. All enrolled patients presented with a history of lumbago, restricted lumbar vertebral function, localized pressing pain, and tenderness upon percussion; magnetic resonance imaging (MRI) revealed the presence of a psoas abscess. The orthopedic surgeons conduct a comprehensive evaluation of each case and collaboratively discuss the treatment plan with the patients and their families based on surgical indications. The criteria for surgery include lesions that compress the spinal cord or nerve roots, resulting in neurological impairment; significant structural damage to the spine (such as vertebral collapse exceeding 50%; vertebral destruction accompanied by intervertebral space narrowing and pedicle destruction leading to spinal instability; and angular deformities exceeding 30°); persistent abscesses existing for more than three months despite anti-tubercular treatment; or the potential for abscess extension into the abdominal cavity, thoracic cavity, or spinal canal, warranting timely intervention. Following diagnosis, all patients received prompt and appropriate anti-tuberculosis chemotherapy, and UPCD of the psoas abscess was performed based on preoperative evaluations and patient preferences. To avoid the impact of multiple lesions requiring repeated treatments on the data, only cases with a single unilateral lesion were included in the study. All patients provided informed consent and were followed up for 24 months. Cases with incomplete data, loss to follow-up, or those who did not accept the treatment in our hospital were excluded. To mitigate the uncertainties associated with treatments administered at external facilities and the resultant bias on outcomes, we excluded cases that had previously undergone treatment at other institutions prior to receiving our intervention. Ultimately, all enrolled cases were categorized into three groups based on the treatment modalities: the surgery-only group, the preoperative UPCD combined with surgery group, and the UPCD-only group.

### 2.2 Equipment and operative techniques

Before the procedure, a comprehensive evaluation of the patients was conducted, including routine blood tests, coagulation profiles, and a history of anticoagulant use. Ultrasound (Philips iU-Elite ultrasonic diagnostic apparatus (Washington, USA) with the C5-1 broadband convex array probe (frequency 1–5 MHz)), CT (Philips Brilliance iCT (Ohio, USA)), and MRI (Philips Achieva (Ohio, USA) 3.0 T) were performed to assess the condition, and personalized management plans were developed based on the results and patient preferences. During the ultrasound examination, the long, short, and height dimensions of the lesion were measured in the longitudinal and transverse axes, and the volume was calculated using the ultrasound’s built-in measurement software.

UPCD-only group: Preoperative planning involved determining the puncture pathway while meticulously avoiding major blood vessels, nerves, and other critical structures. An 8F BIOTEQ pigtail catheter (Taiwan, China) was selected as the drainage tube. After positioning the patient appropriately to fully expose the puncture site, routine disinfection and local anesthesia were performed. Under real-time ultrasound guidance, a one-step puncture technique was employed. Upon reaching the center of the abscess, the trocar stylette was withdrawn, and an attempt was made to aspirate pus. Then the stiffening cannula was removed, and the drainage catheter was placed with a length of 12 ~ 15 cm chosen based on the abscess’s location and size. After ensuring smooth drainage of pus into the drainage bag, the catheter was secured to the skin. The catheter was typically retained for 7 ~ 10 days, with removal contingent upon drainage efficacy and postoperative ultrasound evaluation. Generally, catheter removal was considered when drainage over two consecutive days was less than 2 mL per day, and ultrasound showed no significant residual pus. Anti-tuberculosis chemotherapy continued during this period, with adjustments made according to drug sensitivity tests until the patient’s condition stabilized, meeting discharge criteria. Post-discharge, anti-tuberculosis therapy was continued until clinical cure, with monthly ultrasound follow-ups for 24 months to monitor for recurrence and sinus tract formation.

Preoperative UPCD combined with surgery group: This group included cases where surgery was deemed necessary following evaluation by an orthopedic surgeon. UPCD of TPA was performed preoperatively, as in the UPCD-only group. Surgery was scheduled based on the surgeon’s assessment, with the catheter typically remaining in place for 7 ~ 10 days. Since the surgery not only involved debridement of the abscess and cavity walls but also sequestrum debridement vertebral stabilization, only the duration of abscess debridement and blood loss were recorded for comparison with the surgery-only group. Postoperative care and follow-up were identical to those in the UPCD-only group.

Surgery-only group: This group included patients who required elective surgery, as determined by an orthopedic surgeon, without preoperative UPCD of TPA. The duration of abscess debridement and blood loss were recorded, and postoperative care and follow-up were conducted in the same manner as in the UPCD-only group.

**Figure 1:**
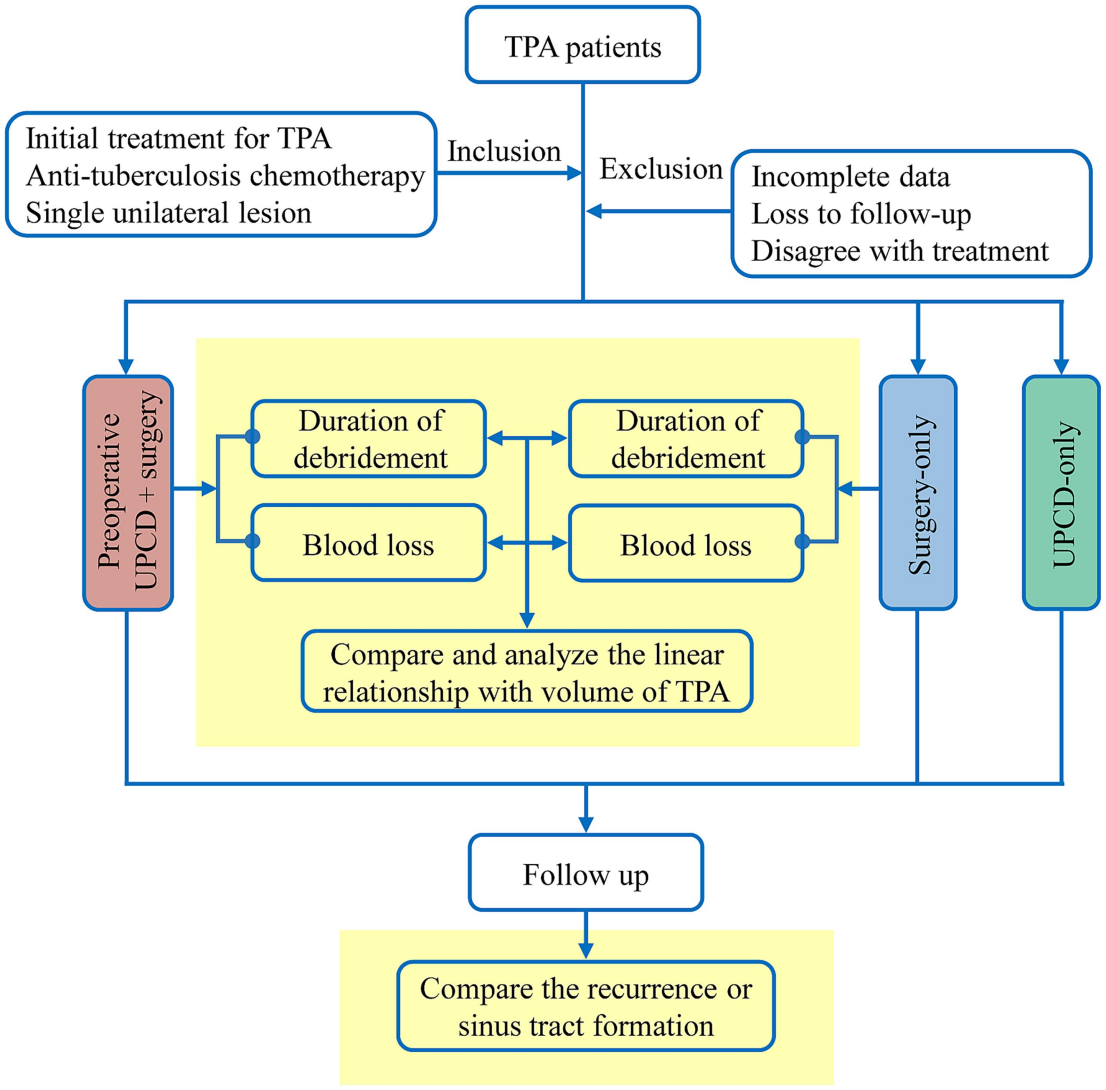
The flowchart. TPA, tuberculous psoas abscesses; UPCD, ultrasound-guided percutaneous catheter drainage.

The procedural workflow is illustrated in Figure 1. A subset of cases in preoperative UPCD combined with surgery group and UPCD-only group were initially diagnosed with TPA at another hospital using the same UPCD method and subsequently presented for treatment at our institution, while the surgeries were conducted by the same team of orthopedic surgeons. All enrolled patients were able to have their all drainage tubes removed prior to discharge, with pain scores recorded at less than 3, significant improvement in neurological compression symptoms, and the majority achieving functional recovery sufficient for independent living.

### 2.3 Statistical analysis

Statistical analysis was performed using the SPSS statistical software package (Version 23.0; SPSS Inc., Chicago, IL). Differences in continuous variables between groups were evaluated using the *t*-test, while categorical data were analyzed using the chi-square test and Fisher’s exact test. A *p* value of less than 0.05 was considered statistically significant. After examining the assumptions of linear regression for each group of data (including normality of residuals, homoscedasticity, et al.), a linear regression analysis was conducted to assess the correlation between abscess volume and debridement duration, as well as intraoperative blood loss, in both the group undergoing preoperative UPCD combined with surgery and the surgery-only group. Kaplan–Meier analysis was employed to assess recurrence and sinus tract formation during the follow-up period across the different groups.

## 3 Results

**Figure 2:**
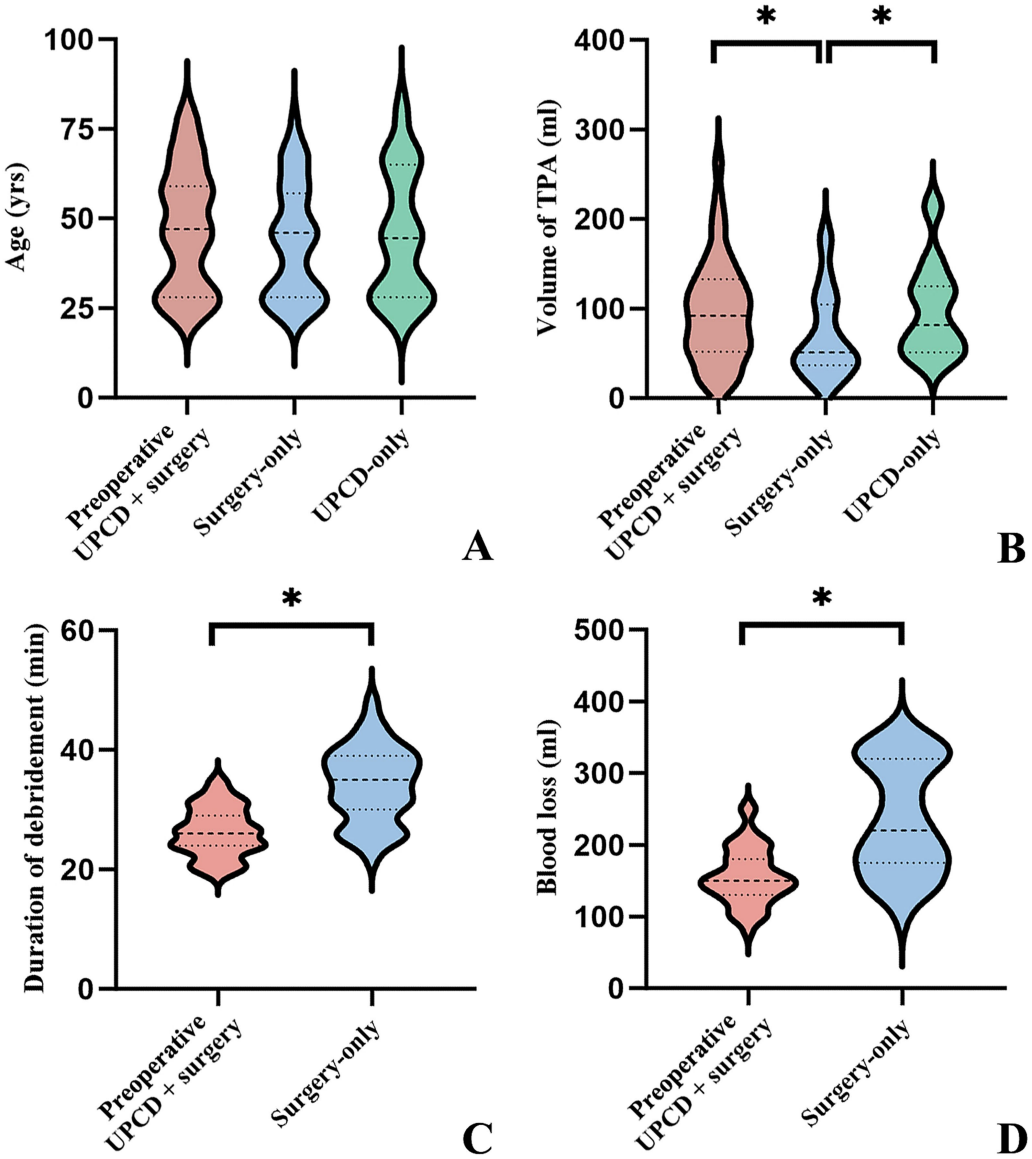
Violin plots of age, TPA volume, debridement duration and blood loss for each group. “*” indicates a statistically significant difference between the two groups. TPA, tuberculous psoas abscesses; UPCD, ultrasound-guided percutaneous catheter drainage.

A total of 114 cases were included in the retrospective study, with basic characteristics detailed in Table 1 and Figure 2. The patients’ ages ranged from 21 to 81 years, with a mean age of 45.56 ± 16.88 years. The surgery-only group included 39 cases, aged 23–77 years (mean 43.56 ± 15.91 years), with 23 males and 16 females. The preoperative UPCD combined with surgery group comprised 43 cases, with an age range of 24–79 years (mean 47.44 ± 17.04 years), including 25 males and 18 females. The UPCD-only group included 32 cases, aged 21–81 years (mean 45.47 ± 18.01 years), with 19 males and 13 females. There were no significant differences in age or gender distribution among the three groups.

**Table 1:** Basic characteristics of the patients and observational parameters across the three groups.

	Surgery-only	Preoperative UPCD + surgery	UPCD-only	*t*/*x*^2^	*p*
Cases	39	43	32		
Mean age (yrs) (M ± SD)	43.56 ± 15.91	47.44 ± 17.04	45.47 ± 18.01		All >0.05
Gender (Female/Male)	16/23	18/25	13/19		All >0.05
Volume of TPA (ml)	70.00 ± 48.20	96.59 ± 56.90*^a^	98.48 ± 52.00*^b^	a: 2.271 b: 2.391	a: 0.026 b: 0.020
Combined with spinal tuberculosis	39	43	5		
Recurrence or sinus tract formation	6	5	5		All >0.05
Duration of debridement (min)	34.18 ± 6.30	26.30 ± 4.14*		6.752	*<*0.001
Blood loss (ml)	237.05 ± 78.18	156.05 ± 39.47*		6.006	<0.001

*: indicates a statistically significant difference when compared to the Surgery-only group.

a: comparison between the preoperative UPCD + Surgery group and the Surgery-only group.

b: comparison between the UPCD-only group and Surgery-only group.

TPA, tuberculous psoas abscesses; UPCD, ultrasound-guided percutaneous catheter drainage.

**Figure 3:**
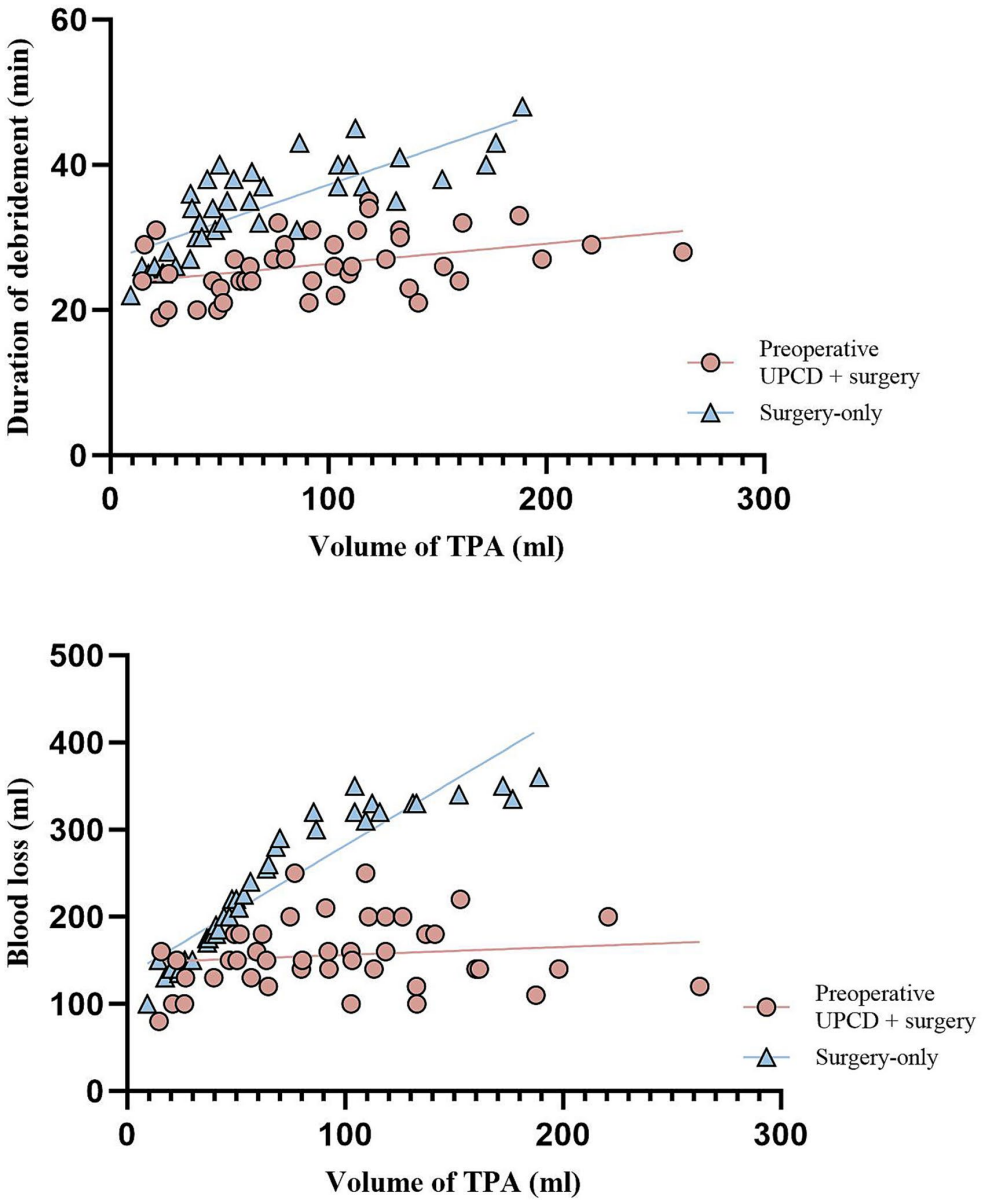
Linear relationship analysis of debridement duration and blood loss with TPA volume. TPA, tuberculous psoas abscesses; UPCD, ultrasound-guided percutaneous catheter drainage.

**Figure 4:**
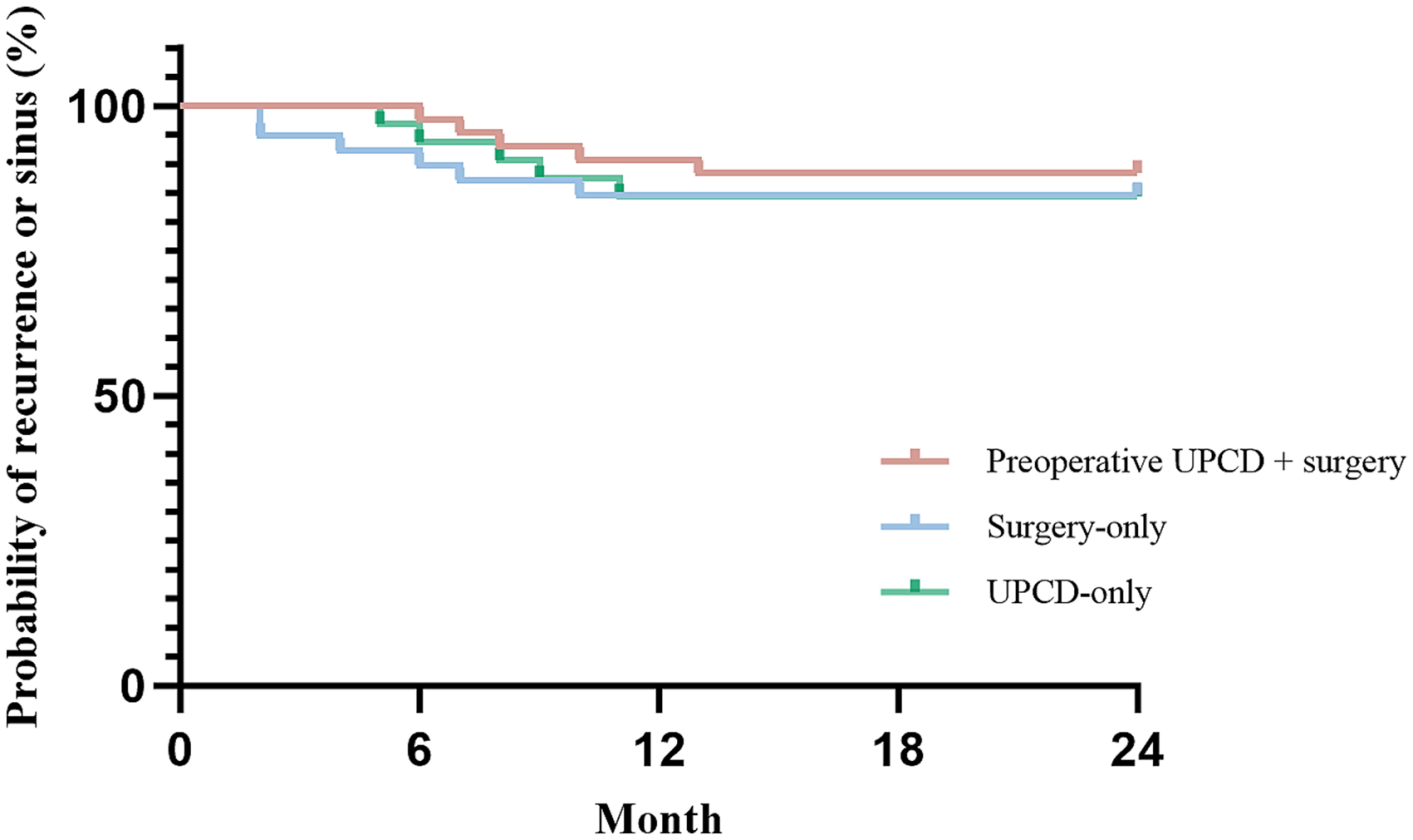
Kaplan–Meier analysis of the probability of recurrence or sinus tract formation in each group. TPA, tuberculous psoas abscesses; UPCD, ultrasound-guided percutaneous catheter drainage.

The mean TPA volume measured by conventional ultrasound was 70.00 ± 48.20 mL (range 9.32–189.00 mL) in the surgery-only group, 96.59 ± 56.90 mL (range 14.55–262.73 mL) in the preoperative UPCD combined with surgery group, and 98.48 ± 52.00 mL (range 33.48–216.06 mL) in the UPCD-only group. Remarkably, the TPA volume in both the preoperative UPCD combined with surgery group and the UPCD-only group was significantly larger compared to the surgery-only group (*t* = 2.271, *p* = 0.026; *t* = 2.391, *p* = 0.020), whereas no significant differences were found between the preoperative UPCD combined with surgery group and the UPCD-only group (*t* = 0.148, *p* = 0.883). A comparison of abscess debridement time and blood loss between the surgery-only group and the preoperative UPCD combined with surgery group was conducted (Tables 1, 2; Figure 3). The abscess debridement time in the preoperative UPCD combined with surgery group was significantly shorter at 26.30 ± 4.14 min, compared to 34.18 ± 6.30 min in the surgery-only group (*t* = 6.752, *p* < 0.001). Blood loss in the preoperative UPCD combined with surgery group was also significantly less, at 156.05 ± 39.47 mL, compared to 237.05 ± 78.18 mL in the surgery-only group (*t* = 6.006, *p* < 0.001). Furthermore, correlation analysis demonstrated a positive relationship between abscess debridement time and TPA volume in both the surgery-only group and the preoperative UPCD combined with surgery group. However, the coefficient B in the preoperative UPCD combined with surgery group (0.028) was markedly lower than that in the surgery-only group (0.103). While blood loss in the surgery-only group was positively correlated with TPA volume (B = 1.494), no significant linear relationship was observed between blood loss and TPA volume in the preoperative UPCD combined with surgery group. Over the 24-month follow-up period, recurrence or sinus tract formation was observed in 5 patients each in the preoperative UPCD combined with surgery group and the UPCD-only group, and in 6 patients in the surgery-only group, with no statistically significant differences detected among the three groups (Figure 4).

**Table 2:** Comparison between preoperative UPCD + surgery group and surgery-only group.

		Surgery-only	Preoperative UPCD + surgery	*t*	*p*
Volume of TPA (ml)		70.00 ± 48.20	96.59 ± 56.90	2.271	0.026
Duration of debridement (min)		34.18 ± 6.30	26.30 ± 4.14	6.752	<0.001
	Linear relationship with volume of TPA	Positive	Positive		
	*R* square	0.620	0.145		
	*F*	60.43	6.961		
	*P*	0.000	0.012		
	Coefficients B	0.103	0.028		
Blood loss (ml)		237.05 ± 78.18	156.05 ± 39.47	6.006	<0.001
	Linear relationship with volume of TPA	Positive	Nonexistent		
	*R* square	0.849	0.173		
	*F*	270.4	0.720		
	*P*	0.000	0.401		
	Coefficients B	1.494	0.091		

TPA, tuberculous psoas abscesses; UPCD, ultrasound-guided percutaneous catheter drainage.

## 4 Discussion

**Figure 5:**
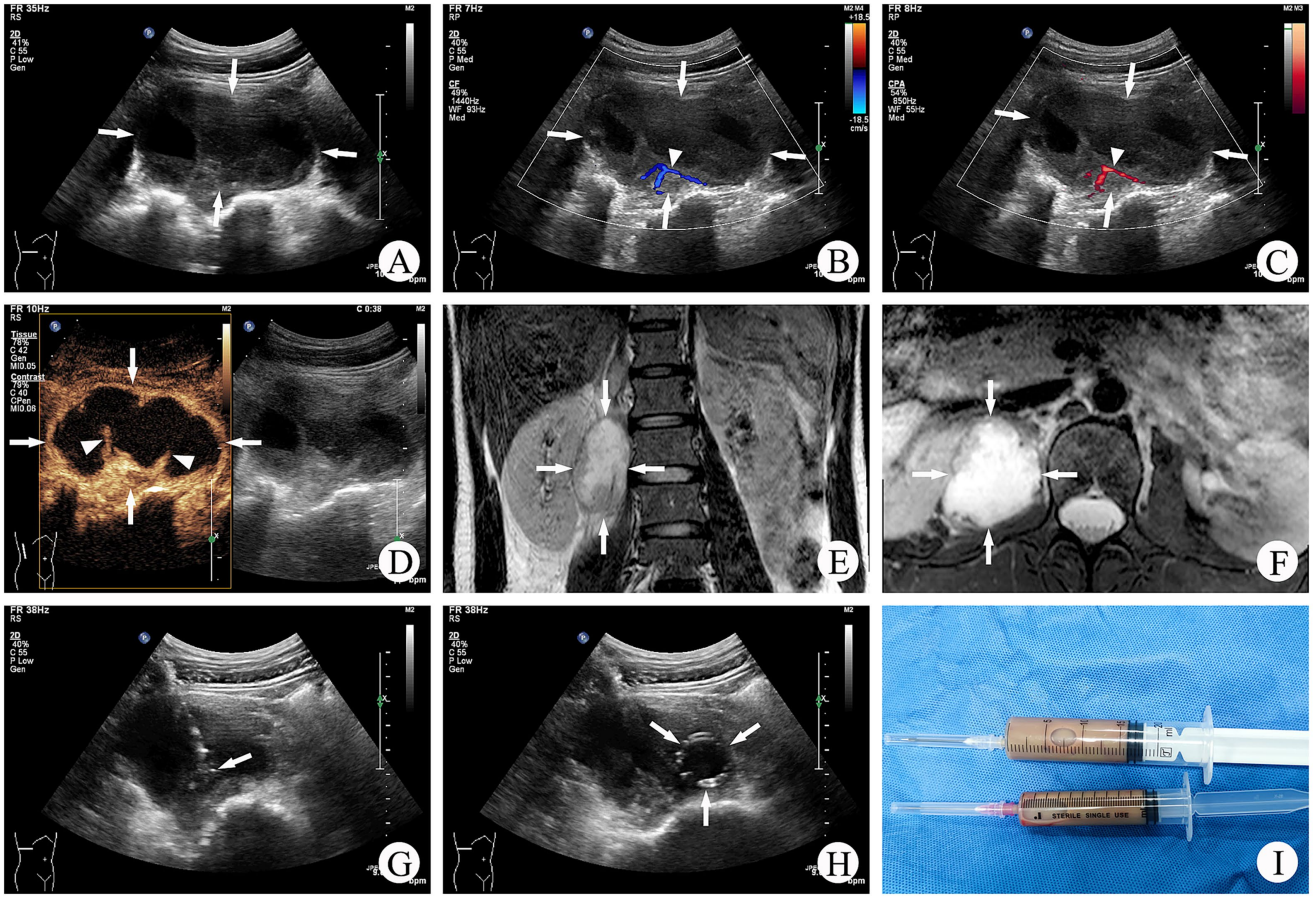
A 34-year-old female patient with a TPA. **(A)** Conventional ultrasound examination revealed a mixed echogenic mass measuring 8.1 × 4.3 cm in the right psoas muscle (↑). **(B,C)** CDFI and CDE indicated a faint signal (△) of blood flow around the mass (↑). **(D)** CEUS demonstrated no enhancement within the mass (↑), with minimal strip-like enhancement observed (△). **(E,F)** MRI showed a patchy area of long T1 and long T2 signals within the right psoas muscle, with scattered punctate signals present (↑). **(G)** Under ultrasound guidance, the tip of the trocar stylette was precisely positioned within the abscess (↑). **(H)** A circularly coiled drainage tube within the abscess (↑). **(I)** The brownish purulent fluid was aspirated, which laboratory analysis confirmed to be a tuberculous abscess.

This study performed a retrospective analysis of three principal treatment modalities for TPA—preoperative UPCD combined with surgery, surgery alone, and UPCD alone—comparing debridement duration, intraoperative blood loss, and rates of recurrence or sinus tract formation. These outcomes, of critical relevance to both clinicians and patients, aim to inform and guide the development of tailored management strategies for TPA. Based on data from our center, UPCD, as a minimally invasive treatment approach, demonstrates distinct advantages whether combined with surgery or administered as a standalone therapy. Consequently, we conducted a comprehensive analysis of its efficacy (Figure 5).

From a pathophysiological perspective, the psoas muscle, characterized by its rich vascular and lymphatic networks, as well as its proximity to the retroperitoneum, exhibits heightened susceptibility to both primary and secondary infections (12). Although psoas abscesses are relatively rare, the escalation of tuberculosis during the 20th century has rendered *Mycobacterium tuberculosis* the predominant etiological agent of psoas abscesses (13–15). Consistent with prior studies, our investigation also identified a higher incidence in males compared to females (6). It is widely acknowledged that primary TPA is associated with immunosuppression, whereas secondary TPA typically arises from direct spread or hematogenous or lymphatic dissemination from adjacent infected organs or tissues, most commonly stemming from spinal tuberculosis (Pott’s disease) (8, 16–20). In this study, 110 cases (75.3%) were associated with concomitant spinal tuberculosis. Currently, the standard management for TPA entails early, combined, appropriately dosed, and consistent anti-tuberculosis chemotherapy administered throughout the entirety of the treatment course (5). Nevertheless, the role of surgical debridement remains a subject of ongoing debate (9, 10). Our orthopedic experts generally deemed surgical intervention necessary in the presence of indications such as neurological impairment and significant structural damage to the spine (21–23). During the surgical procedure, the surgeons were tasked not only with debriding the abscess and its cavity walls but also with excising the sequestrum and stabilizing the compromised skeletal and joint structures. With the advancement of imaging technologies, image-guided abscess drainage has emerged as a prominent alternative to conventional surgical intervention (24). Ultrasound, in particular, is extensively employed in both diagnosis and treatment due to its real-time capabilities, cost-effectiveness, convenience, and absence of ionizing radiation (25). The ultrasonic characteristics of TPA have been thoroughly documented in the literature and need not be reiterated herein (26–28). Reports suggest that UPCD offers significant advantages, including minimal dissemination of purulent material and a reduced systemic inflammatory response, along with the added benefit of being feasible under local anesthesia (24, 29).

To ensure comparability between the preoperative UPCD combined with surgery group and the surgery-only group in this study, we exclusively documented the duration of abscess debridement and the associated blood loss, deliberately excluding the time allocated for bone and joint stabilization from the analysis. In our study, the preoperative UPCD combined with surgical intervention demonstrated notably shorter debridement times and decreased blood loss compared to the surgery-only group, despite the larger abscess volumes observed in the former. This outcome may be attributed to a substantial reduction in abscess cavity pressure following preoperative drainage, which leads to diminished compression of surrounding tissues, attenuated inflammatory responses, and reduced granulation tissue formation within adjacent soft tissues. Furthermore, upon drainage of the purulent fluid, the abscess wall collapses due to loss of structural support, subsequently contracting and separating from adjacent tissues (30). Through these combined mechanisms, more efficient surgical dissection and debridement of the abscess wall are facilitated (12, 31). Surgeons had identified that the most time-consuming aspect of abscess debridement was the separation and removal of the abscess cavity wall (32). Therefore, preoperative drainage can significantly reduce the duration of surgical debridement. Correlation analysis revealed that while debridement time exhibited a positive correlation with abscess volume in both groups, the coefficient B was significantly higher in the surgery-only group. This indicates that larger abscesses required more debridement time in the surgery-only group. Simultaneously, our linear regression analysis indicated that the R-squared value for the surgery-only group exceeded that of the preoperative UPCD combined with surgery group, signifying a superior fit between abscess volume and debridement duration in the surgery-only group, thus providing an alternative interpretation of the preceding analysis. Additionally, we noted a linear positive correlation between debridement blood loss and abscess volume in the surgery-only group, whereas no such relationship was observed in the preoperative UPCD combined with surgery group. This indicates that larger abscesses in the surgery-only group resulted in greater blood loss during debridement, while blood loss in the preoperative UPCD combined with surgery group appeared to be independent of abscess volume. This phenomenon may be attributed to the decreased pressure within the abscess cavity following preoperative drainage, leading to diminished granulation tissue formation, reduced neovascularization, and an overall tendency for surrounding tissues to recuperate under the influence of repair factors (8, 33). Consequently, the cavity walls of the abscess contracted and shrank as the cavity became smaller and emptier, resulting in reduced irritation to the body and allowing absorption to surpass exudation, thereby leading to a more manageable lesion (8, 32). This, in turn, facilitates the separation of the abscess wall from the surrounding normal soft tissues and minimizes hemorrhage. On the other hand, the preoperative abscess drainage facilitates easier separation of the abscess wall, thereby enhancing surgical efficiency and consequently reducing intraoperative blood loss.

Furthermore, some patients, following a comprehensive evaluation by surgeons, who exhibited stable conditions or minimal vertebral involvement, were managed exclusively with abscess drainage. Over a 24-month follow-up period, the drainage-only group demonstrated comparably favorable outcomes, with no statistically significant differences in recurrence or sinus tract formation rates when juxtaposed with the preoperative UPCD combined with surgery group and the surgery-only group. Kaplan–Meier analysis of the follow-up results across the three groups also revealed no statistically significant differences. This indicates that UPCD of TPA can achieve curative results akin to those of surgical intervention when accompanied by consistent anti-tuberculosis chemotherapy. The underlying rationale was that the management of TPA primarily relies on anti-tubercular chemotherapy, which effectively eradicates *Mycobacterium tuberculosis* or inhibits its growth and proliferation (5). Concurrently, the principal objectives of abscess management are to attenuate the body’s response to the abscess, prevent further invasion of surrounding tissues and organs, and promptly evacuate purulent material from regions where drug concentrations are insufficient to achieve therapeutic levels, thereby facilitating the body’s recovery (24). Although debridement surgery can most effectively accomplish these objectives, its associated surgical trauma and complications may impede the patient’s healing process (10). However, for patients exhibiting severe vertebral destruction and instability, surgical stabilization remains imperative (21–23).

In summary, UPCD plays a pivotal role in the management of TPA. For patients with TPA complicated by severe vertebral involvement and instability, preoperative UPCD can significantly reduce surgical duration and intraoperative blood loss, thereby mitigating surgical complications and promoting expedited recovery. Furthermore, regarding long-term outcomes, patients with minimal or absent vertebral involvement who undergo UPCD in conjunction with sustained anti-tubercular chemotherapy may achieve therapeutic results comparable to those attained through surgical debridement.

Naturally, this study was not without its limitations. Firstly, the variability in surgical approached contingent upon the patient’s condition introduced a degree of bias when broadly estimating debridement time and blood loss. Moreover, this study primarily concentrated on readily observable statistical outcomes such as debridement time, blood loss, and rates of recurrence or sinus tract formation, while neglecting the statistical analysis of pain scores, functional outcomes, neurological assessments, and other relevant metrics. Secondly, patient-specific factors, including overall health status and resistance to anti-tuberculosis medications, could influence outcomes. Thirdly, treatment decisions in this study were predicated on patient conditions rather than complete randomization, potentially leading to selection bias. The subsequent collection of additional pertinent data would facilitate multivariate regression analysis and propensity score matching, thereby significantly enhancing the robustness of the study. Furthermore, this study utilized data from a single tuberculosis diagnosis and treatment center, and its data on TPA incidence and technical treatment capabilities may not be fully representative of all medical institutions. Additionally, the limited sample size for certain data points may impact the validity of the results. These issues underscore the necessity for further comprehensive research.

## 5 Conclusion

In conclusion, UPCD of TPA demonstrates significant therapeutic efficacy. This technique offers a minimally invasive management option in clinical practice and facilitates the provision of more personalized therapeutic approaches for patients.

## Data Availability

The original contributions presented in the study are included in the article/supplementary material, further inquiries can be directed to the corresponding author.
